# Interpretation of Genetic Association Studies: Markers with Replicated Highly Significant Odds Ratios May Be Poor Classifiers

**DOI:** 10.1371/journal.pgen.1000337

**Published:** 2009-02-06

**Authors:** Johanna Jakobsdottir, Michael B. Gorin, Yvette P. Conley, Robert E. Ferrell, Daniel E. Weeks

**Affiliations:** 1 Department of Biostatistics, Graduate School of Public Health, University of Pittsburgh, Pittsburgh, Pennsylvania, United States of America; 2 Department of Ophthalmology and Jules Stein Eye Institute, The David Geffen School of Medicine, University of California Los Angeles, Los Angeles, California, United States of America; 3 Department of Health Promotion and Development, School of Nursing, University of Pittsburgh, Pittsburgh, Pennsylvania, United States of America; 4 Department of Human Genetics, Graduate School of Public Health, University of Pittsburgh, Pittsburgh, Pennsylvania, United States of America; University of Michigan, United States of America

## Abstract

Recent successful discoveries of potentially causal single nucleotide polymorphisms (SNPs) for complex diseases hold great promise, and commercialization of genomics in personalized medicine has already begun. The hope is that genetic testing will benefit patients and their families, and encourage positive lifestyle changes and guide clinical decisions. However, for many complex diseases, it is arguable whether the era of genomics in personalized medicine is here yet. We focus on the clinical validity of genetic testing with an emphasis on two popular statistical methods for evaluating markers. The two methods, logistic regression and receiver operating characteristic (ROC) curve analysis, are applied to our age-related macular degeneration dataset. By using an additive model of the *CFH*, *LOC387715*, and *C2* variants, the odds ratios are 2.9, 3.4, and 0.4, with *p*-values of 10^−13^, 10^−13^, and 10^−3^, respectively. The area under the ROC curve (AUC) is 0.79, but assuming prevalences of 15%, 5.5%, and 1.5% (which are realistic for age groups 80 y, 65 y, and 40 y and older, respectively), only 30%, 12%, and 3% of the group classified as high risk are cases. Additionally, we present examples for four other diseases for which strongly associated variants have been discovered. In type 2 diabetes, our classification model of 12 SNPs has an AUC of only 0.64, and two SNPs achieve an AUC of only 0.56 for prostate cancer. Nine SNPs were not sufficient to improve the discrimination power over that of nongenetic predictors for risk of cardiovascular events. Finally, in Crohn's disease, a model of five SNPs, one with a quite low odds ratio of 0.26, has an AUC of only 0.66. Our analyses and examples show that strong association, although very valuable for establishing etiological hypotheses, does not guarantee effective discrimination between cases and controls. The scientific community should be cautious to avoid overstating the value of association findings in terms of personalized medicine before their time.

## Introduction

Recent successes in the discoveries of potentially causal single nucleotide polymorphisms (SNPs) for complex diseases hold great promise, and commercialization of genomics in personalized medicine has already begun. A number of companies now offer, for relatively modest fees, personalized genomics services that provide individualized disease-risk estimates based on genome-wide SNP genotyping. Most companies offering such profiling make it clear that they are not a clinical service and that their calculations are not intended for diagnostic or prognostic purposes. They typically advise their clients to consult their health care provider for more information. In most cases, people would turn to their general physician [1]. However, as noted by others [2],[3], few doctors currently have enough genetics training to actually make sense of the risk calculations now commercially offered. Many physicians seem to feel the same way. In surveys in five European countries, physicians ranked the disciplines in which they felt they needed more training to overcome future challenges [4],[5]. In all countries, the top ranked area was “genetics of common disease,” and ranked second was “approaching genetic risk assessment in clinical practice.”

Not only are risk results likely to be often poorly understood by the tested individuals and their physicians, but also these results are often based on risk models, such as logistic regression models, that may not be good classification models [6]. Therefore, the disclaimer made by the companies that their services are not intended as medical advice cannot be overemphasized. Current knowledge of the role of most genes in complex diseases is at the group level of correlations of disease status with SNPs. Most of these SNPs were discovered via genetic association studies aimed at finding variants correlated with disease risk. It is hoped that these discoveries will provide insights into the pathogenesis and etiology, and ultimately lead to developments of new treatments or preventive therapies. Assuming these SNPs will also be effective classifiers, they are now being used in individual-level risk estimation, classification, and clinical decision-making. However, for many complex diseases, such as the ones discussed here (age-related macular degeneration [AMD], type II diabetes, inflammatory bowel disease [Crohn's disease], and cardiovascular disease), it is arguable whether the era of genomics in personalized medicine is here yet. In this article, we discuss and explore how useful highly associated SNPs might be for individual-level risk estimation and prediction. Our focus will be on the classification accuracy of genetic testing, with an emphasis on two popular statistical methods for evaluating biomarkers. We give realistic real-data examples that illustrate that, currently, the genetic information is of limited value for personalized medicine. We also discuss and apply risk-based and classification-based analysis approaches to our AMD data.

## Two Statistical Methods

There are two basic statistical approaches for evaluating markers. The risk-based approach models the risk as a function of marker(s), often with adjustment for covariates, and is commonly applied in genetic studies. In case-control studies, this is done with logistic regression, and the markers with the strongest effect on disease risk are those associated with the smallest *p*-values and most extreme odds ratios (ORs). The other method, the classification-based approach, evaluates markers based on how well they can discriminate between cases and controls. The performance is evaluated by various measures, such as the proportion of positive test results among cases or the true positive fraction (TPF, or sensitivity) and the proportion of positive test results among controls or the false positive fraction (FPF, or 1−specificity). A perfect classifier will assign a positive test result to everyone with the condition (TPF = sensitivity = 1) and a negative test result to everyone without the condition (FPF = 0, specificity = 1). Often more than one possible grouping into cases and controls is possible based on a classifier. The receiver operating characteristic (ROC) curve is a plot of all (FPF, TPF) pairs for each possible grouping. The area under the ROC curve (AUC) is a popular measure of the discrimination power of a classifier. It is the probability that given two random individuals, one who will develop the disease and the other who will not, the classifier will assign the former a positive test result and the latter a negative result. Theoretically, the AUC can take values between 0 and 1, but the practical lower bound is 0.5; a perfect classifier has an AUC of 1. Classifiers with an AUC significantly greater than 0.5 have at least some ability to discriminate between cases and controls. However, for screening of individuals with an increased risk of disease, it is suggested that the AUC be >0.75, and for presymptomatic diagnosis of the general population, the AUC should be >0.99 [7]. When prognosis is the goal, one typically also evaluates the classification model by two additional measures: (1) the proportion of individuals who will develop the disease among those with a positive test result, or the positive predictive value (PPV), and (2) the proportion of individuals who will not develop the disease among those with negative test result, or the negative predictive value (NPV) (Box 1). We note in passing that there are other methods that model classification performance and have been applied in genetic studies, including, for example, genetic algorithms, generalized multifactor dimensionality reduction, and random forests [8]–[10]. However, to keep our discussion focused, we do not discuss these other methods here.

**Box 1. Classification performance measures—definitions**

TPF = probability that a diagnostic test (e.g., a marker or a risk model) classifies an individual as a case given that this person is truly affected = *P*(test positive | affected)

FPF = probability that a diagnostic test classifies an individual as a case given that the person is actually unaffected (a control) = *P*(test positive | unaffected).

PPV = probability that a person who tests positive is actually a case = *P*(affected | test positive)

NPV = probability that a person who tests negative is actually a control = *P*(unaffected | test negative)

Although the risk-based (logistic regression) and classification-based (ROC theory) methods do not yield contradictory results in terms of directionality, they can and often will differ in terms of size or importance. For example, a marker strongly related to risk may very well be a poor classifier; and vice versa, a good classifier may only be weakly associated with risk [6]. Furthermore, neither method directly measures calibration, which is how well the predicted risks agree with the underlying true risks [11] (Box 2).

**Box 2. Association versus classification versus risk prediction and calibration**

Strong association (low *p*-value) does not guarantee effective discrimination between cases and controls (classification). Excellent classification (high AUC) does not guarantee good prediction of actual risk. A model that accurately predicts risk is well calibrated.

In a diagnostic setting in which discrimination between cases and controls is most important, it only matters that the cases have higher estimated risk, accurate or not, than the controls. However, when prognosis or risk stratification is the goal, both discrimination and calibration are important. We then need a model that both discriminates well between future cases and those who will remain controls, and also accurately estimates the exact risk of developing disease in the future.

## The Odds Ratio, Classification, Calibration, and Prediction

The OR is widely used to evaluate markers, and it is assumed the markers associated with the most extreme OR are effective predictors. However, as we mentioned above, a marker strongly related to risk may very well be a poor classifier, and vice versa, a good classifier may only be weakly associated with risk [6]. In addition, a marker associated with risk may be well or poorly calibrated, that is, the predicted risk may agree well or poorly with the true risk [11].

**Figure 1:**
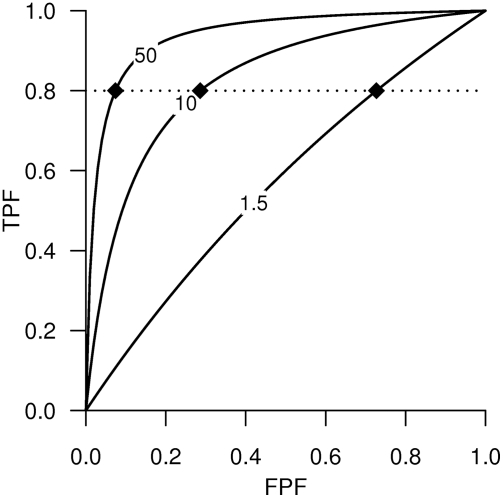
Accuracy curves for binary markers. The curves of accuracy points (FPF, TPF pair) for binary markers with ORs 1.5, 10, and 50 are plotted. The black diamonds and horizontal dotted line highlight the points (FPF, TPF) = (FPF, 80%) on the accuracy curves. The ORs are marked on the curves.

For a strongly associated marker to be effective in classification, the associated OR must be of an extreme magnitude rarely (if ever) seen in genetic association studies. As illustrated in Figure 1, if one wants to be able to detect 80% of cases with a binary marker, such as the presence or absence of a risk allele, with ORs of 1.5, 10, or 50, then about 73%, 29%, and 7% of the controls would be mislabeled as cases, and the AUC achieved by the binary marker would be 0.54, 0.76, and 0.86, respectively. Even a huge OR of 50 does not guarantee that a marker will have acceptable prediction accuracy; for example, the TPF may be unacceptably low (TPF = 55%, FPF = 2.4%, and AUC = 0.76) or the FPF unacceptably high (TPF = 97.6%, FPF = 45%, and AUC = 0.76) (Figure 1).

**Figure 2:**
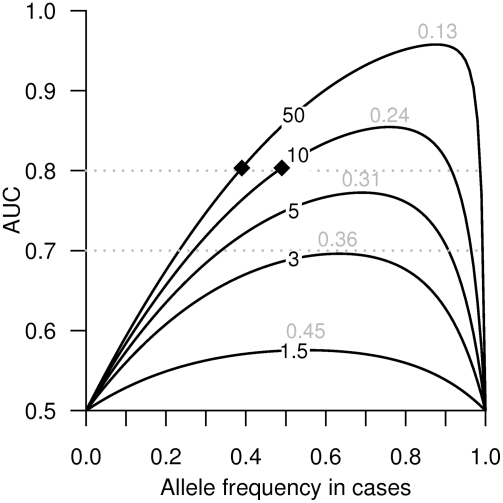
AUC for additive risk models of SNP markers as function of risk allele frequency in cases. The AUC is estimated for all risk allele frequencies in controls assuming additive ORs 1.5, 3, 5, 10, and 50 (the ORs are marked on the curves). The numbers in gray are the risk allele frequencies in controls corresponding to the maximum AUC for each OR. The dotted horizontal line in gray marks an AUC of 0.7 and 0.8. The black diamonds highlight the points (*p*_ca_, AUC) = (*p*_ca_, 0.80) for markers with additive ORs 10 and 50 (see Table 1).

Let us examine the achievable AUC as a function of risk allele frequency under an additive genetic model in which the genotypes are coded 0, 1, and 2 (Figure 2 and Table 1). In Figure 2, we have plotted the AUC for fixed values of the OR, as a function of risk allele frequency in cases (*p*_ca_) under the assumption of Hardy-Weinberg equilibrium in both cases and controls. We clearly see that markers with a reasonably high OR of 3 have a maximum possible AUC of less than 0.70, and markers with an OR of 5 do not even reach an AUC of 0.80. For each OR, the risk allele frequency in controls (*p*_co_) corresponding to the maximum possible AUC is given on the plot, and not surprisingly, to reach the maximum possible AUC for each OR, the risk allele frequency difference between cases and controls has to be quite large (Table 1). For example, to reach an AUC of 0.80 using a marker with an OR of 10, the allele frequencies in cases and controls would be quite different (*p*_ca_ = 0.49 and *p*_co_ = 0.09) (Table 1).

**Table 1:** AUC, Risk Allele Frequencies in Cases (*p*_ca_) and Controls (*p*_co_) for Specific ORs in an Additive Model (Genotypes Coded 0-1-2 According to Number of Risk Alleles).

OR	Maximum AUC	*p*_ca_	*p*_co_	AUC = 0.80	
				*p*_ca_	*p*_co_
1.5	0.58	0.55	0.45	NP	NP
3	0.70	0.63	0.36	NP	NP
5	0.77	0.69	0.31	NP	NP
10	0.85	0.76	0.24	0.49	0.09
50	0.96	0.88	0.13	0.39	0.01

NP, not possible.

## The Odds Ratio, Relative Risk, and Risk

In retrospective studies, the relative risk or risk ratio (RR) cannot be estimated unless the prevalence is known, and therefore, the OR is used as a proxy. Theoretically, the OR will give a good approximation for the RR if the prevalence is low, but otherwise it tends to overestimate the RR [12],[13]. RRs, which are the ratio of two risks (probabilities), are correctly interpreted as an estimate of how much more likely people sharing the same genotype combination are to develop the condition of interest when compared to a group without this genotype combination. The numerator of the RR is the risk of the condition given the genotype combination of interest, but clearly, the RR (or the OR) itself is not an estimate of individual-level risk and certainly not a diagnostic test or classifier.

Statisticians should easily understand this relationship between OR, RR, and risk, but a person not trained in statistics (or science in general) may not make the same distinction as easily. Numerous studies in the genetic counseling literature have investigated what people make of risk estimates. For example, in a study of women's perceived risk of breast cancer, 98% of women overestimated their risk of dying from breast cancer in 10 y by half to 8-fold when asked to quantify risk as a number out of 1,000. Interestingly, only 10% of those women thought they were at higher risk than an average woman their age [14].

## Clinical Validity and Utility of Predictive Genetic Testing

The clinical validity is measured by the discrimination ability of the marker, or its ability to classify people as cases or controls. The AUC, though imperfect, is a popular and easily interpretable measure of classification accuracy. It can be interpreted as the probability that predicted risk is higher for a case than a control. Various TPF and FPF pairs and various values of the AUC can correspond to the same OR (Figure 1). Thus, the OR by itself cannot give a meaningful indication of the probability of being correctly classified as case (TPF) or of the probability of being wrongly classified as a case (FPF), and alone its value is essentially useless to the individual.

The clinical utility of predictive genetic profiling for complex diseases rests on at least two conditions: (1) preventive means with high efficacy in the general population are available, and (2) these preventive means will also be effective in the genetically high-risk cohorts. Additionally, it is worth noting that for many complex diseases, known preventive lifestyle changes are broadly beneficial: weight loss, smoking cessation, blood pressure control, regular exercise, diets enriched with fruits and vegetables, etc., so to many individuals, it might be wasteful to spend $1,000 to find out they are genetically at increased risk for some condition only to have their doctor tell them all they can do is to lose weight and stop smoking. On the other hand, if the person is more likely to make lifestyle changes and stick to them, then the benefits can be great, both for the individual and the population as whole. Of course, the flip side is what the actions will be if the genetic test suggests lower than average risk for one or more specific conditions.

## Reclassification

The AUC attempts to measure the ability of a model to discriminate between cases and controls for a set of cutoff values that separate the two groups. However, on an individual basis, we also want the model to provide the best possible estimation of that person's risk. One way to compare the accuracy of individual-level risk estimates of different risk models is to use the reclassification table approach [11],[15]. In this approach, one measures how often subjects are estimated to be in different risk strata when different risk models are applied and whether the reclassification more accurately stratifies individuals into higher or lower risk strata. A marker that has a modest or no effect on the AUC can improve risk classification [11]. For example, suppose we are comparing two risk models that differ regarding a single individual's membership in the 20%–30% risk stratum versus the 10%–20% risk stratum. If both models achieve the best discrimination by classifying everyone below the 40% risk threshold as controls and everyone above as cases, then the TPF and FPF will not be altered due to this person's reclassification, but one model is more accurate than the other in terms of the true value of the individual's risk estimate.

## Examples

We now provide several examples, from the literature as well as from our own data, illustrating that although a set of SNPs can be strongly associated with disease risk with extremely small *p*-values, that same set of SNPs may not necessarily have high discrimination ability or may not dramatically improve the discrimination ability of a classification model constructed using “conventional” nongenetic risk factors without the SNPs.

### Risk of Cardiovascular Events

In a recent replication study of nine SNPs associated with levels of either low-density lipoprotein (LDL) or high-density lipoprotein (HDL) cholesterol, Kathiresan et al. [16] created a genotype score on the basis of the total number of unfavorable alleles at these risk SNPs, and investigated the classification accuracy of the genotype score and the effect on reclassification beyond standard risk factors for cardiovascular events. The authors found that accounting for the effect of the nine SNPs did not improve the classification accuracy of their model. The ROC curves with and without the genotype score lined up almost perfectly, and both had an AUC of 0.80 despite the SNPs having *p*-values as low as 10^−29^, with six out of nine SNPs having *p*-values<10^−6^ (Text S1 and Table S1 in Text S1). Adding the genotype score to the model did, however, modestly improve the reclassification. Unfortunately for this dataset, the classification accuracy of the genotype score alone was not estimated. Nevertheless, these data provide an example of highly associated variants that do not markedly improve the discrimination ability of a model, yet at the same time, they give hope that genetic variants may become valuable prognostic tools.

### Risk of Type 2 Diabetes

In type 2 diabetes, 12 SNPs [17]–[19] with *p*-values as low as 10^−34^ (Text S1 and Table S2 in Text S1) reach an AUC of 0.64, suggesting only fair discrimination power. We arrived at this AUC of 0.64 using only published allele frequencies; we did this using the method of Lu and Elston [20] (Text S1, Estimating the AUC from meta-data). Lu and Elston [20] also applied their method to a model of the same 12 SNPs and four additional environmental factors, and got a slightly improved AUC of 0.67.

### Risk of Prostate Cancer

A genetic classification model of two prostate cancer risk SNPs in low linkage disequilibrium with each other [21] has an AUC of 0.56, based on the method of Lu and Elston [20]. An AUC of this magnitude suggests that the model has a very poor discrimination power. The SNPs have *p*-values of 10^−13^ and 10^−14^, but the genotype-specific ORs are not extreme and range from 1.3 to 2.2 (Text S1 and Table S3 in Text S1).

### Risk of Inflammatory Bowel Disease

A genetic classification model of five well-replicated genetic associations [22]–[26] in inflammatory bowel disease (Crohn's disease) has an AUC of only 0.66. This suggests only fair discrimination power for Crohn's disease despite the variants being highly significant (*p*-values range from 10^−7^ to 10^−14^) and one SNP having quite an extreme OR of 0.26 (∼1/4). Again, the method of Lu and Elston was used to estimate the AUC [20]. For more details, see Text S1 and Table S4 in Text S1.

### Risk of Age-Related Macular Degeneration

**Figure 3:**
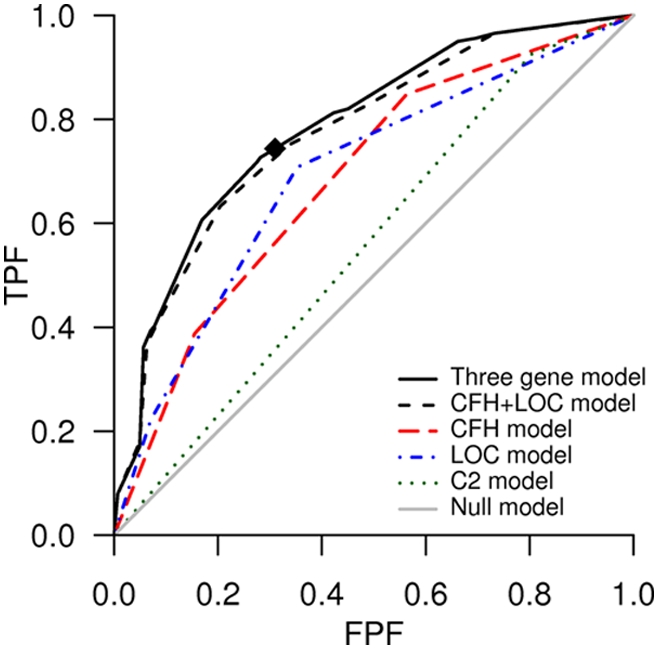
ROC curves for AMD classification models. The black diamond highlights the point (FPF, TPF) = (31%, 74%) on the ROC curve of the three-factor model of *CFH*, *LOC387715*, and *C2*. The gray line for reference gives the “chance” classification rule: the farther the ROC curve is from the chance line, the better the classification rule.

Using our previous published AMD data [10] on the *CFH*, *LOC387715*, and *C2* variants, we plotted the ROC curves and estimated the AUC and positive predictive values of one-, two-, and three-factor models (detailed methods are in Text S1). Figure 3 displays the ROC curves for the null model and for five genetic risk models: the three-factor model of *CFH*, *LOC387715*, and *C2* SNPs, the two-factor model of *CFH* and *LOC387715*, and all of the one-factor models. We see that to correctly identify about 74% of the cases using the three-factor model, we would wrongly classify 31% of the controls, and for the TPF to be around 80%, the FPF needs to be unacceptably high (>40%). The AUC for the three-factor model is quite high, 0.79, and significantly different from 0.5 (95% confidence interval [CI] 0.74–0.83) (Table 2). Table 2 also gives the results of logistic regression analysis: the ORs for additive inheritance of *CHF* and *LOC387715* risk alleles are about 3 with *p*-values of around 10^−13^.

**Table 2:** Results of Logistic Regression and ROC Analysis.

Model	Factors	Logistic Regression		ROC Analysis	
		OR	*p*-Value	AUC	95% CI
**Model 1**	*CFH*	2.89	9.1×10^−13^	0.79	0.74–0.83
	*LOC387715*	3.42	2.3×10^−13^	0.79	0.74–0.83
	*C2*	0.39	1.3×10^−3^	0.79	0.74–0.83
**Model 2**	*CFH*	3.00	9.1×10^−14^	0.77	0.73–0.82
	*LOC387715*	3.38	2.5×10^−13^	0.77	0.73–0.82
**Model 3**	*CFH*	2.77	2.1×10^−13^	0.69	0.64–0.73
**Model 4**	*LOC387715*	3.11	6.2×10^−13^	0.69	0.65–0.74
**Model 5**	*C2*	0.33	1.9×10^−5^	0.56	0.53–0.60

The OR for each variant is for an additive model in which the genotypes are coded 0-1-2.

The confidence intervals (CIs) for the AUC are asymptotic and derived using DeLong's estimator [48] for the variance.

**Figure 4:**
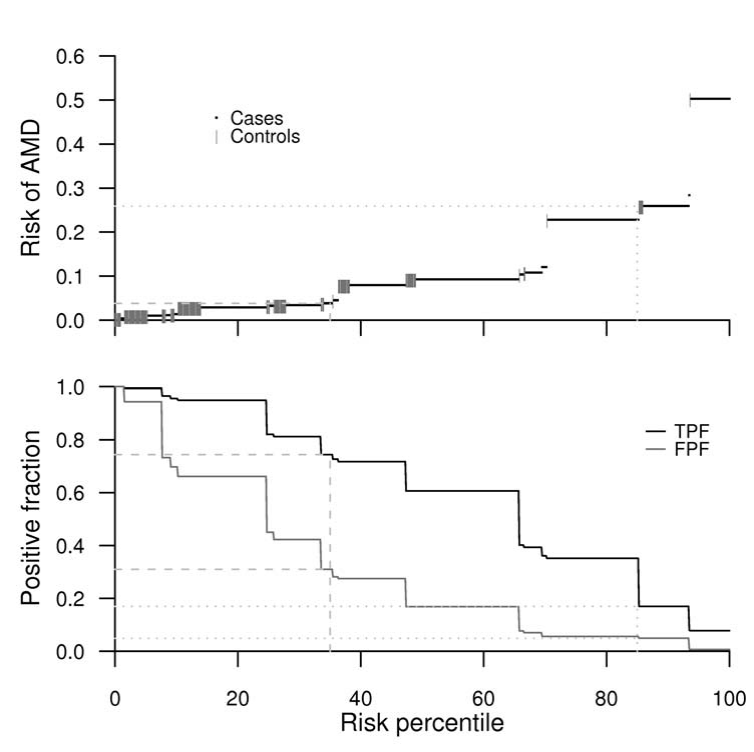
Integrated predictiveness and classification plot for the three-factor model. The light-gray lines show how the plots are used in the examples given in the text: the dashed lines are for the first example with TPF = 74%, FPF = 31%, risk percentile = 35%, and AMD risk threshold = 4%; and the dotted lines are for the second example with AMD risk threshold = 25%, risk percentile = 85%, TPF = 17%, and FPF = 5%. On the top panel, the risks for cases are marked with a dot in black while the risks for controls are marked with a vertical line segment in dark-gray.

We also plotted the integrated predictiveness and classification plot, which combines information from both the risk- and classification-based analysis approaches discussed above [27]. In the integrated plot (Figure 4), there are two aligned plots: in the top plot, ordered individual risks are plotted as function of the risk percentile, and in the bottom plot, the TPF and FPF are plotted as a function of the risk percentile such that at each point, the TPF and FPF are calculated for the risk threshold equal to the risk associated with the corresponding risk percentile. If we now look at the integrated predictiveness and classification plot for the three-factor model, we see that the TPF and FPF pair 74% and 31% corresponds to the 35% risk percentile (Figure 4, bottom panel), which then corresponds to choosing an AMD risk of 4% as the cutoff point for classifying individuals (Figure 4, top panel). Those with risk greater than 4% are assumed to be at high risk and are classified as cases, and those with lower risk are classified as controls. To illustrate this, suppose we have a population of size 1,000 and a prevalence of 5.5% (which is the prevalence of advanced AMD in the U.S. in white individuals 65 y or older according to Friedman et al. [28] and the U.S. 2000 census data—see Text S1 for further details). If the prevalence is 5.5%, there would be 55 cases in our population. Of those 55 cases, 74%, or 41, would be correctly considered to be at high risk of AMD, and 31%, or 293, of the true 945 controls would be wrongly assumed to be at high risk. Therefore, out of the 334 (41+293) individuals in the high-risk group, 88% should actually be in the low-risk group, or in other words, the PPV would be only 12% (i.e., 100%−88%). When designing a clinical trial to test preventive therapies in high-risk cohorts based on genotyping alone, it may or may not be cost effective to have 12% (instead of 5.5%) of the study cohort as true cases. However, as a clinical test, it may be considered unethical to needlessly alarm 88% of the high-risk cohort, especially when limited treatment and preventive options are available [29].

To lower the proportion of controls in the high-risk cohort, a more stringent threshold for calling someone high risk, say 25%, can be used instead of the 4% threshold used above. However, using this higher risk threshold only lowers the proportion of controls in the high-risk group from 88% to 84%, as can be seen in this manner: the plot (Figure 4, top panel) shows that the risk threshold of 25% corresponds to the 85% risk percentile. Looking at the classification curve (Figure 4, bottom panel), we see that the 85% risk percentile corresponds to a TPF of 17% and FPF of 5%. Again, to put these numbers in perspective, let us again assume we have a population of size 1,000. Nine (17%) out of 55 true cases would then be correctly classified as high risk, and 47 (5%) out of 945 controls would be incorrectly classified as high risk. Therefore 84% (47/56 = 47/(9+47)) of those classified as “high risk” would actually be controls (PPV = 100%−84% = 16%).

When applied to case-control data, the integrated predictiveness and classification plot depends on the assumed prevalence of the disease, which may not be known with precision or may, as in the case of AMD, depend strongly on age. Note that as the prevalence changes, the bottom plot does not change, only the top plot does: although it still will look essentially the same, the risks will be more spread out between 0 and 1 as the prevalence gets higher and less spread out otherwise.

Second, it is worth noting how the results of our AMD example change if different values for the prevalence are used. The prevalence of AMD is highly age-dependent, and in Table 3, we calculate the PPV using prevalence estimates for different age groups. If the prevalence increases, the results are less disappointing (PPV increases) but are even worse if it decreases (Table 3). Clearly, the ability to discriminate between current cases and controls, based on genotype data from *CFH*, *LOC387715*, and *C2* alone, changes with age. A crude estimate of the lifetime risk at age 80 y, given a genetically high-risk score based on the three variants, is 30% compared to 15% baseline lifetime risk at age 80 (Table 3).

**Table 3:** Positive Predictive Values (PPVs) for Different Values of the Prevalence.

Prevalence	Age Group	Risk Threshold	PPV
15%	80 y and older	10%	30%
5.5%	65 y and older	4%	12%
1.5%	40 y and older	1%	3%

The risk threshold corresponds to TPF = 74% and FPF = 31% (as in the first example in the text).

PPV = proportion of cases in the high-risk group.

1−PPV = proportion of controls in the high-risk group.

## Discussion of the AMD Example

If the primary goal of genetic diagnostic tests for AMD were to identify those who are at high risk before they show irreversible degenerative changes to maximize the effectiveness of long-term preventive strategies, then we would want to test individuals 40–55 y old (or younger) to predict whether they will develop AMD before age 80 y. Our case-control data presented here do not fully measure the ability of genetic data to predict future disease status (prognosis) for several reasons: (1) AMD prevalence increases with age, (2) females have higher prevalence in all age groups compared to males, (3) females live longer, (4) the FPF derived from case-control data is overestimated because some controls will develop AMD as the cohort ages, (5) the case/control counts are unbalanced, so our sample may not be optimal for estimating the classification accuracy of the markers [30], and (6) the estimates of the ORs, and estimates from most other AMD case-control studies, are based on the comparison of extreme phenotypes: a group of individuals with advanced AMD are contrasted with a control group of individuals with no or very minimal clinical findings. Therefore, they very likely overestimate the RR and the discrimination power for individuals with intermediate clinical findings. Even accounting for all these issues in an optimistic manner, the overall conclusions of our analysis are unlikely to change dramatically. Proper analyses of longitudinal cohort data using survival analysis techniques could lead to a more precise assessment of the potential value of genetic data in predicting lifetime AMD status [31],[32].

The major achievements that have been made in understanding the genetics of AMD are well known, and the AMD discoveries [33]–[38] are widely mentioned as the first “proof” that genome-wide association analysis works (although the majority of the AMD studies were not genome-wide association studies, but rather targeted searches following up regions of linkage). The results have been so exciting that perhaps all of us who study AMD are guilty of overstating our results. Here are just a few examples:

“Nevertheless, with all the genetic findings, it may soon be possible to provide pre-symptomatic diagnosis with reasonable accuracy, leading to better disease management strategies for high-risk individuals.”—Swaroop et al. [39]

“The continued support for these genes in ARM susceptibility will hopefully bring us closer to being able to utilize the information in these genes to identify at risk individuals and provide a rational basis for future clinical trials to test preventive therapies in high-risk cohorts.”—Conley et al. [40]

“Expressed another way, these genotypes apparently identify individuals whose lifetime risk of AMD ranges from less than 1% to more than 50%; however, longitudinal studies are needed to define the true risk attributable to these loci and the ways in which these might interact with the known environmental and lifestyle risk factors.”—Maller et al. [41]

All these statements are scientifically valid, they are carefully worded, and it is clear the investigators are talking about “potential,” “future,” and “hope.” Nevertheless, they can and have been overinterpreted. For example, a recent review [42] cites Maller et al. [41] and states:

“SNPs in complement factor H (*CFH*) and *PLEKHA1/ARMS2/HtrA1* capture a substantial fraction of AMD risk and permit the identification of individuals at high risk of developing AMD.”

Even *Nature Genetics* appears to also overstate the potential impact of AMD genetics. In the December 2007 issue [43], the editors discuss the new hype about personalized genomics and ask: “With the possible exception of age-related macular degeneration, how much can we say with confidence about the spectrum of risk?” However, as we have shown here, we cannot yet make an exception for AMD. We should, however, not let this discourage us. The discoveries of the AMD risk genes are truly amazing, and they should of course encourage and guide future research. In fact, the discovery of the likely involvement of the *CFH* gene gave firmer footing to the hypothesis that the abnormal function of complement pathway can cause AMD and has resulted in discoveries of other AMD genes in this pathway [9], [44]–[46].

## Conclusions

Genetic association studies have identified many susceptibility variants for complex diseases and, in many cases, added to the understanding of the etiology of the diseases. However, as we discuss here using real data and theoretical examples, strong association does not necessarily guarantee good classification or discrimination ability. Before using association results for classification and risk estimation purposes, we need to establish their effectiveness formally using appropriate measures and, ideally, appropriate study designs. Additionally, when evaluating the improvement in the predictive value by adding a marker to a prediction model, we may need to use additional measures besides the AUC, such as reclassification tables.

In our examples, we saw that the addition of nine highly significant risk SNPs to the risk model could not improve the discrimination power for cardiovascular events beyond standard risk factors. For type 2 diabetes, the classification rule based on 12 SNPs gave an AUC of only 0.64, a value that is well below the guidelines of 0.75 and 0.99 cutoffs for screening and prognosis purposes, respectively. For Crohn's disease, a classification model based on five SNPs gave an AUC of only 0.66, and for prostate cancer, a model of two SNPs achieves an AUC of only 0.56. Both values are well below the 0.75 and 0.99 cutoffs. For AMD, the AUC of a model with three SNPs was 0.80, but the proportion of positive test results among affected individuals was only 30%, 12%, and 3%, depending on assumed prevalence (15%, 5.5%, and 1.5%, respectively). The results of these four examples, although somewhat disappointing, are not surprising given the theoretical results of Janssens et al. [7],[47] that indicate that achieving a high AUC requires a much larger number of genetic variants than we have to date. For example, Janssens et al. demonstrated that for genetic profiling, on average 80 common variants with ORs of 1.25 each were needed to develop a model useful for identification of high-risk individuals (AUC>0.80).

Even though our examples illustrate that highly associated SNPs may not be effective as classifiers, it should not be concluded that the association findings are not important nor that association studies are not valuable. In many cases, the association discoveries have and will continue to result in new etiological hypotheses previously not considered. For example, in the case of AMD, the *CFH* discovery [33]–[35],[37] resulted in a new focus on the complement pathway and subsequent identification of additional novel disease genes in that pathway [9], [44]–[46]. The scientific community should be very cautious to avoid overhyping association findings in terms of their “personalized medicine” value before their time, lest we lose the goodwill and support of the general public.

## Supporting Information

Text S1 Supporting text and tables.
(0.08 MB PDF)
