# Supplementary material for: Interpretation of Genetic Association Studies: Markers with Replicated Highly Significant Odds Ratios May Be Poor Classifiers
Source: PLoS Genet. 2009 Feb 6;5(2):e1000337. doi: 10.1371/journal.pgen.1000337 (PMC2629574; doi:10.1371/journal.pgen.1000337)
Supplement: Text S1 — Supporting text and tables. (0.08 MB PDF) [file pgen.1000337.s001.pdf]

Text S1

Application of classification-based methods to AMD data

AMD is a complex, late-onset degenerative disease that is characterized by the disruption of the integrity of the retina, retinal pigment epithelium and choroid that can lead to the loss of central vision and significant visual disability. In recent years, three loci have been found strongly associated with AMD: functional SNPs at CFH [1-3] and LOC387715 (or at the closely linked PLEKAH1 [MIM 607772] or HTRA1 [MIM 602194]) [4,5] are thought to increase the risk of AMD while variants at CFB (MIM 138470) or C2 (MIM 217000) [6] are thought to decrease risk. These findings appear robust and have been widely replicated [7]. A number of studies have attempted to use variants at these genes to build predictive models for AMD. To our knowledge, none has applied ROC theory to evaluate the classification performance of these variants individually or jointly.

AMD data

For illustration we use part of our AMD data, which includes 640 cases and 142 controls fully typed at three SNPs at all three loci: rs1061170 (Y402H) in CFH, rs10490924 (S69A) in LOC387715, and rs547154 (IVS10) in C2. For recruitment and phenotyping we refer to our previous publications [8,9] and for genotyping see [10].

Methods

To combine information from the three SNPs for classification using ROC, we use a generalized linear model proposed by Ma and Huang [11]: $P(Y=1|\mathbf{X}) = G(ß^T\mathbf{X})$, where Y is the disease status (Y=1 for cases and Y=0 for controls), $\mathbf{X}$ is the matrix of genotype columns $X_i = (X_{1,i}, \ldots, X_{d,i})^T$ for the ith subject, $ß = (ß_1, \ldots, ß_d)^T$ is a d-dimensional vector of unknown regression parameters, and G is an unknown increasing link function. Since G is assumed to be increasing, a classification rule can be constructed based on the risk score, $ß^T\mathbf{X}$, only. We use the rule: if $ß^T\mathbf{X} > c$, we classify this individual as a case, otherwise we classify the individual as a control. This is a sensible approach as decision criteria based on risk are statistically optimal [12]. The overall performance of the classifier is then measured by the AUC of the ROC curve, which is a two-dimensional plot of $((FPF(c), TPF(c)): c \in \mathbf{R})$, where FPF(c), and TFP(c) are the FPF and TPF of the classification rule if $ß^T\mathbf{X} > c$. To get all points on the ROC curve the FPF and TPF are estimated for all possible values of c. The empirical AUC is maximized as a function of ß. For each ß, the AUC is estimated using a nonparametric trapezoidal estimator [13]. Note that this ROC model is a more general model than the logistic model, as G needs not to be known. Since many previous studies have found an additive model to be best fitting in both single and multi locus models of CFH, LOC387715, and C2 variants, we let $X_{1,i}$ be the number of risk alleles at Y402H at CFH, $X_{2,i}$ be the number of risk alleles at S69A at LOC387715, and $X_{3,i}$ be the number of protective alleles at IVS10 at C2. For comparison, we also present the results of logistic regression analyses where the genotypes are coded the same way.

In addition to performing ROC and logistic regression analyses, we draw an integrated predictiveness and classification plot [14]. In the integrated plot, there are two aligned plots: In the top plot, ordered individual risks are plotted as function of the risk

percentile and, in the bottom plot, the TPF and FPF are plotted as a function of the risk percentile such that at each point the TPF and FPF are calculated for the risk threshold, c, equal to the risk associated with the corresponding risk percentile. As we are working with case-control data, we can only calculate individual-level risks from the logistic model (setting the G function to be the logit function) if the prevalence is known. To be able to draw the plot we therefore need to assume a specific value for the prevalence. Since our data are elderly white individuals (mean age 72.9 and standard deviation [sd] 9.9 in controls) and our cases are all of advanced phenotype, we use a prevalence estimate for advanced AMD in white individuals 65 years and older (approximately 1 sd from the mean) of 5.5%; the US 2000 census data (Table 4: Annual Estimates of the White Alone Population by Age and Sex for the United States: April 1, 2000 to July 1, 2006 [NC-EST2006-04-WA]) were used to project the sex-specific 5-year age interval estimates of Friedman et al. [15] to estimate the AMD prevalence for 65 years and older.

Accounting for covariates

We also ran the above analysis while adjusted for age, sex, and smoking. The AUC of the genetic model without the covariates was 0.78 and improved to 0.82 when the covariates were added to the model. The AUC of model with only the covariates had an AUC of 0.66. Note that the effective sample size for these new analyses is smaller due to missing covariate information. The AUC of the unadjusted model in the main text (0.79) is therefore, not exactly equal to the AUC of the unadjusted model here (0.78).

Estimating the AUC from meta-data

As science progresses, there is a need for methods to continuously update previous classification models. Lu and Elston [16] developed a method to do this when only meta-data and summary statistics are available. This is especially useful if not all markers have been typed in the same samples. Then, if we assume homogeneity across samples, we can combine estimates to form a new classification rule. To compare the AUC of the new classification rule with the old rule, the information we need are 1) allele frequencies in case and control populations or 2) allele frequencies in the general population, risk ratios, and prevalence.

Details on data in other real data examples

Cardiovascular events

Kathiresan et al. [17] investigated whether genetic variants could improve classification accuracy for cardiovascular events beyond standard risk factors. First they tested for single SNP associations of 11 SNPs with low-density lipoprotein (LDL) and high-density lipoprotein (HDL) levels and then identified a set of 9 SNPs that were independently associated with lipid levels. Using these 9 SNPs, they created a simple genotype score based on the total number of unfavorable alleles in all 9 genotypes of the individual, and then evaluated the classification accuracy of the genotype score for the 10-year incidence of cardiovascular events. The p-values for the 9 SNPs ranged from 0.003 to 10^{-29} (Table S1) and the adjusted hazard ratio of the genotype score was 1.15 (95% CI 1.07-1.24).

Table S1 Association results of 9 SNPs associated with LDL and HDL cholesterol. Information from Table 2 of Kathiresan et al. [17]

SNP	P-value
**LDL cholesterol**	2×10^{-11}
rs693	8×10^{-7}
rs4420638	3×10^{-21}
rs12654264	0.002
rs1529729	0.003
rs11591147	7×10^{-7}
**HDL cholesterol**	
rs3890182	0.003
rs1800775	2×10^{-29}
rs1800588	4×10^{-10}
rs328	3×10^{-12}

The AUC for prediction of 10-year incidence of cardiovascular events was estimated using model with 14 clinical covariates and no genotype information and found to be 0.80. When the genotype score, which included several highly associated SNPs, was included in the model, the AUC was not improved and also equaled 0.80 even though accounting for the genotype score significantly improved the regression model (P-value 0.0002, Table S3 of Kathiresan et al [17]). The authors additionally looked at whether accounting for the genotype score improved the clinical reclassification and found modest improvement such that the estimated risk correctly increased for individuals who subsequently experienced cardiovascular event and correctly decreased for individuals who remained free of cardiovascular events at 10-year follow-up (P-value 0.01).

Type 2 diabetes

The 12 SNPs used to generate a classification rule for type 2 diabetes with the Lu and Elston method [16] come from three studies (Table S2) [18-20].

Table S2 Association results of 12 type 2 diabetes SNPs

SNP	Allele frequency in cases	Allele frequency in controls	P-value	OR	Study
rs5219	0.384	0.354	0.0001	1.14	Weedon [20]
rs1801282	0.099	0.123	4×10^{-5}	1.29	Weedon [20]
rs7903146	0.406	0.293	2×10^{-34}	Het 1.65, Hom 2.77	Sladek [19]
rs13266634	0.254	0.301	6×10^{-8}	Het 1.18, Hom 1.53	Sladek [19]
rs1111875	0.358	0.402	3×10^{-6}	Het 1.19, Hom 1.44	Sladek [19]
rs740010	0.336	0.301	1×10^{-4}	Het 1.14, Hom 1.40	Sladek [19]
rs3740878	0.240	0.272	1×10^{-4}	Het 1.26, Hom 1.46	Sladek [19]
rs4402960	0.341	0.304	8×10^{-4}	1.18	Scott [18]
rs7754840	0.387	0.360	0.0095	1.12	Scott [18]
rs10811661	0.872	0.850	0.0022	1.20	Scott [18]
rs9300039	0.924	0.892	7×10^{-8}	1.49	Scott [18]
rs8050136	0.406	0.381	0.017	1.11	Scott [18]

Information from the combined cohort of stage and 2 used from the Scott et al. study.

Prostate cancer
We used the Lu and Elston method [16] to investigate the classification accuracy of a genetic risk model of two prostate cancer risk SNPs [21] (Table S3). We used the information from the combined cohort from the study of Yeager et al. [21].

Table S3 Association results of two prostate cancer disease SNPs

SNP	Allele frequency in cases	Allele frequency in controls	P-value	OR_{het}	OR_{hom}
rs1447295	0.15	0.11	2×10^{-14}	1.43	2.23
rs6983267	0.56	0.50	9×10^{-13}	1.26	1.58

Inflammatory bowel diseases
We used the Lu and Elston method [16] to investigate the classification accuracy of genetic risk model of five SNPs. Two SNPs are in IL23R and are thought to be uncorrelated, one in ATG16CL, one in NOD2/CARD15, and one in IRGM; all are associated with Crohn's disease (which is a form of inflammatory bowel disease) (Table S4).

Table S4 Association results of five Crohn's disease SNPs

SNP	Allele frequency in cases	Allele frequency in controls	P-value	OR	Study
rs11209026	0.019	0.070	5×10^{-9}	0.26	Duerr et al. [22]
rs751784	0.345	0.448	5×10^{-9}	0.89	Cummings et al. [23]
rs2241800	0.61	0.52	2×10^{-7}	1.45	Cummings et al. [24]
rs2076756	0.358	0.244	7×10^{-14}	NA	Rioux, et al. [25]
rs13361189	0.098	0.067	4×10^{-8}	1.38	Parkes et al. [26]

References

1. Edwards AO, Ritter R, 3rd, Abel KJ, Manning A, Panhuysen C, et al. (2005) Complement factor H polymorphism and age-related macular degeneration. Science 308: 421-424.
2. Haines JL, Hauser MA, Schmidt S, Scott WK, Olson LM, et al. (2005) Complement factor H variant increases the risk of age-related macular degeneration. Science 308: 419-421.
3. Klein RJ, Zeiss C, Chew EY, Tsai JY, Sackler RS, et al. (2005) Complement factor H polymorphism in age-related macular degeneration. Science 308: 385-389.
4. Jakobsdottir J, Conley YP, Weeks DE, Mah TS, Ferrell RE, et al. (2005) Susceptibility genes for age-related maculopathy on chromosome 10q26. Am J Hum Genet 77: 389-407.
5. Rivera A, Fisher SA, Fritsche LG, Keilhauer CN, Lichtner P, et al. (2005) Hypothetical LOC387715 is a second major susceptibility gene for age-related macular degeneration, contributing independently of complement factor H to disease risk. Hum Mol Genet 14: 3227-3236.
6. Gold B, Merriam JE, Zernant J, Hancox LS, Taiber AJ, et al. (2006) Variation in factor B (BF) and complement component 2 (C2) genes is associated with age-related macular degeneration. Nat Genet 38: 458-462.
7. Gorin MB (2007) A clinician's view of the molecular genetics of age-related maculopathy. Arch Ophthalmol 125: 21-29.
8. Weeks DE, Conley YP, Mah TS, Paul TO, Morse L, et al. (2000) A full genome scan for age-related maculopathy. Hum Mol Genet 9: 1329-1349.
9. Weeks DE, Conley YP, Tsai HJ, Mah TS, Schmidt S, et al. (2004) Age-related maculopathy: a genomewide scan with continued evidence of susceptibility loci within the 1q31, 10q26, and 17q25 regions. Am J Hum Genet 75: 174-189.
10. Jakobsdottir J, Conley YP, Weeks DE, Ferrell RE, Gorin MB (2007) C2 and CFB Genes in Age-related Maculopathy and Joint Action with CFH and LOC387715 Genes. Submitted.
11. Ma S, Huang J (2007) Combining multiple markers for classification using ROC. Biometrics 63: 751-757.
12. McIntosh MW, Pepe MS (2002) Combining several screening tests: optimality of the risk score. Biometrics 58: 657-664.
13. Zhou XH, Obuchowski NA, McClish DK (2002) Statistical Methods in Diagnostic Medicine. New York: John Wiley & Sons, Inc.
14. Pepe MS, Feng Z, Huang Y, Longton G, Prentice R, et al. (2007) Integrating the Predictiveness of a Marker with Its Performance as a Classifier. Am J Epidemiol.
15. Friedman DS, O'Colmain BJ, Munoz B, Tomany SC, McCarty C, et al. (2004) Prevalence of age-related macular degeneration in the United States. Arch Ophthalmol 122: 564-572.
16. Lu Q, Elston RC (2008) Using the optimal receiver operating characteristic curve to design a predictive genetic test, exemplified with type 2 diabetes. Am J Hum Genet 82: 641-651.
17. Kathiresan S, Melander O, Anevski D, Guiducci C, Burtt NP, et al. (2008) Polymorphisms associated with cholesterol and risk of cardiovascular events. N Engl J Med 358: 1240-1249.

18. Scott LJ, Mohlke KL, Bonnycastle LL, Willer CJ, Li Y, et al. (2007) A genome-wide association study of type 2 diabetes in Finns detects multiple susceptibility variants. Science 316: 1341-1345.
19. Sladek R, Rocheleau G, Rung J, Dina C, Shen L, et al. (2007) A genome-wide association study identifies novel risk loci for type 2 diabetes. Nature 445: 881-885.
20. Weedon MN, McCarthy MI, Hitman G, Walker M, Groves CJ, et al. (2006) Combining information from common type 2 diabetes risk polymorphisms improves disease prediction. PLoS Med 3: e374.
21. Yeager M, Orr N, Hayes RB, Jacobs KB, Kraft P, et al. (2007) Genome-wide association study of prostate cancer identifies a second risk locus at 8q24. Nat Genet 39: 645-649.
22. Duerr RH, Taylor KD, Brant SR, Rioux JD, Silverberg MS, et al. (2006) A genome-wide association study identifies IL23R as an inflammatory bowel disease gene. Science 314: 1461-1463.
23. Cummings JR, Ahmad T, Geremia A, Beckly J, Cooney R, et al. (2007) Contribution of the novel inflammatory bowel disease gene IL23R to disease susceptibility and phenotype. Inflamm Bowel Dis 13: 1063-1068.
24. Cummings JR, Cooney R, Pathan S, Anderson CA, Barrett JC, et al. (2007) Confirmation of the role of ATG16L1 as a Crohn's disease susceptibility gene. Inflamm Bowel Dis 13: 941-946.
25. Rioux JD, Xavier RJ, Taylor KD, Silverberg MS, Goyette P, et al. (2007) Genome-wide association study identifies new susceptibility loci for Crohn disease and implicates autophagy in disease pathogenesis. Nat Genet 39: 596-604.
26. Parkes M, Barrett JC, Prescott NJ, Tremelling M, Anderson CA, et al. (2007) Sequence variants in the autophagy gene IRGM and multiple other replicating loci contribute to Crohn's disease susceptibility. Nat Genet 39: 830-832.
